# Transcriptomic responses to drought stress in *Polygonatum kingianum* tuber

**DOI:** 10.1186/s12870-021-03297-8

**Published:** 2021-11-15

**Authors:** Huali Qian, Zhe Xu, Kun Cong, Xinyan Zhu, Lei Zhang, Junfeng Wang, Jiankun Wei, Pengzhang Ji

**Affiliations:** 1 School of Chinese Materia Medica and Yunnan Key Laboratory of Southern Medicinal Resource, Yunnan University of Chinese Medicine, Kunming, 650500 China; 2 Institute of Medicinal Plants, Yunnan Academy of Agricultural science, Kunming, 650223 China

**Keywords:** Carotenoid biosynthesis, Gingerol, Medicinal plant, Polysaccharides, Secondary metabolites, Starch and sucrose biosynthesis, Semi-arid, Water stress

## Abstract

**Background:**

*Polygonatum kingianum* Coll. et Hemsl. is an important plant in Traditional Chinese Medicine. The extracts from its tubers are rich in polysaccharides and other metabolites such as saponins. It is a well-known concept that growing medicinal plants in semi-arid (or drought stress) increases their natural compounds concentrations. This study was conducted to explore the morpho-physiological responses of *P. kingianum* plants and transcriptomic signatures of *P. kingianum* tubers exposed to mild, moderate, and severe drought and rewatering.

**Results:**

The stress effects on the morpho-physiological parameters were dependent on the intensity of the drought stress. The leaf area, relative water content, chlorophyll content, and shoot fresh weight decreased whereas electrolyte leakage increased with increase in drought stress intensity. A total of 53,081 unigenes were obtained; 59% of which were annotated. We observed that 1352 and 350 core genes were differentially expressed in drought and rewatering, respectively. Drought stress driven differentially expressed genes (DEGs) were enriched in phenylpropanoid biosynthesis, flavonoid biosynthesis, starch and sucrose metabolism, and stilbenoid diarylheptanoid and gingerol biosynthesis, and carotenoid biosynthesis pathways. Pathways such as plant-pathogen interaction and galactose metabolism were differentially regulated between severe drought and rewatering. Drought reduced the expression of lignin, gingerol, and flavonoid biosynthesis related genes and rewatering recovered the tubers from stress by increasing the expression of the genes. Increased expression of carotenoid biosynthesis pathway related genes under drought suggested their important role in stress endurance. An increase in starch and sucrose biosynthesis was evident from transcriptomic changes under drought stress. Rewatering recovered the drought affected tubers as evident from the contrasting expression profiles of genes related to these pathways. *P. kingianum* tuber experiences an increased biosynthesis of sucrose, starch, and carotenoid under drought stress. Drought decreases the flavonoids, phenylpropanoids, gingerol, and lignin biosynthesis. These changes can be reversed by rewatering the *P. kingianum* plants.

**Conclusions:**

These results provide a transcriptome resource for *P. kingianum* and expands the knowledge on the effect of drought and rewatering on important pathways. This study also provides a large number of candidate genes that could be manipulated for drought stress tolerance and managing the polysaccharide and secondary metabolites’ contents in *P. kingianum.*

**Supplementary Information:**

The online version contains supplementary material available at 10.1186/s12870-021-03297-8.

## Background

*Polygonatum kingianum* Coll. et Hemsl. is one of the 71 species included in the family Liliaceae and is grown at the elevation of 700–3600 m. In China, it is distributed in Yunnan, Sichuan, Guangxi, and Guizhou provinces; it has generally a southern distribution [1, 2]. The Polygonatum species are particularly confined to northern hemisphere and they inhabit parts of England and move eastwards to Japan, China, and eastern Siberia [2]. In the Traditional Chinese Medicine system, the rhizome of *P. kingianum* is frequently used for the treatment of muscle fatigue, feebleness, osteoporosis, spleen qi deficiency, stomach yin deficiency, dry cough, blood deficiency, age-related diseases, and diabetes owing to the presence of bioactive compounds [3]. The other *Polygonatum* species are used in drinks, snacks, vegetables, staple food, and seasoning in China [4]. The most commonly used part of this plant is the rhizome (Huangjiang in Chinese) [5]. The extracts from *P. kingianum* rhizome are abundant in alkaloids, flavones, steroid saponins, lignins, amino acids, and polysaccharides [6–9]. Particularly, the presence of polysaccharides and saponins has attained higher attention in recent years in the health industry [10, 11].

It is a well-known concept that medicinal plants grown under semi-arid conditions result in an increased concentration of medicinally important natural products [12]. The environment of semi-arid and arid subtropics sometimes includes both limited water supplies and increased radiation, which together impose drought stress on the plants. The drought conditions lead towards changes in biosynthesis and accumulation of secondary metabolites [13]. The drought stress driven increased secondary metabolite accumulation is a common feature but at the same time, plants exhibit reduced growth and biomass accumulation [14]. Nevertheless, species and even genotypes differ in their responses to biotic and abiotic stresses. For example, a literature survey conducted to understand the effect of drought stress on both quality and concentration variation of secondary metabolites (particularly essential oils) reported that drought causes an overall decrease in essential oil content [15].

Drought stress impairs different morphological, physiological, and biochemical traits in plants. Visible effects can be seen by observing the morphology of the plants. Most commonly, it has been reported that drought stress significantly reduces the leaf area, and causes early maturity as well as changes in root and shoot length [16]. The reduction in leaf area is directly linked with the chlorophyll content and photosynthetic efficiency [17, 18]. The reduced chlorophyll content causes yellowness in the leaves. Additionally, plants experiencing drought also express the effect of imposed stress in the form of reduction in relative water content [19]. Together with reduced water content, the deficiency in chlorophyll and reduced photosynthesis is accompanied by the accumulation of reactive oxygen species (ROS), which leads to cell death when the drought stress is prolonged. The extent of cell death can be evaluated by determining the electrolyte leakage [20]. In tuber crops like potato, earlier studies have concluded that such morpho-physiological traits greatly affect the carbon portioning and starch accumulation in tubers under drought stress conditions [21, 22]. Thus, a similar morpho-physiological response may be expected in the *P. kingianum* plants and it is possible that the starch and sucrose metabolism and related pathways might be affected.

Advancements in genomics have enabled the large-scale transcriptome profiling of essential genetic resources that are of commercial and medicinal importance. Researchers are increasingly exploring the species with medicinal importance to understand the effect of drought on secondary metabolites and other related pathways. Recently, transcriptome analyses of *Capparis spinosa*, *Ammopiptanthus mongolicus*, *Eleusine coracana*, and *Opisthopappus taihangensis* explored the effect of drought on plant and/or on the biosynthesis of polysaccharides and secondary metabolites [23–26]. Similarly, the transcriptome profiles of *Polygonatum sibiricum*, and *P. kingianum* have aided in the discovery of genes involved in polysaccharide and steroidal saponins biosynthesis [27, 28]. However, to our knowledge, there are no reports on the exploration of the transcriptomic response of *P. kingianum* tubers in response to drought stress and rewatering. Considering the medicinal importance of *P. kingianum*, the effect of drought stress tolerance on the essential pathways related to polysaccharides and secondary metabolites is highly needed. This knowledge will answer the questions if *P. kingianum* is affected by drought in similar ways as the widely accepted concept of increasing natural component concentration under stress conditions?

In this study, we have explored the transcriptomic response of *P. kingianum* Coll. et Hemsl. which is a red-flower bearing genotype, captures 70% of *P. kingianum* market, and cultivated over 4000 ha in China. We compared the transcriptome of *P. kingianum* tubers grown under mild, moderate, and severe drought. We also compared the transcriptomic response of drought-affected *P. kingianum* tubers with the rewatered tubers and explored the differentially expressed genes and pathways.

## Results

### Morpho-physiological responses of *P. kingianum* plants to drought and rewatering

**Fig. 1:**
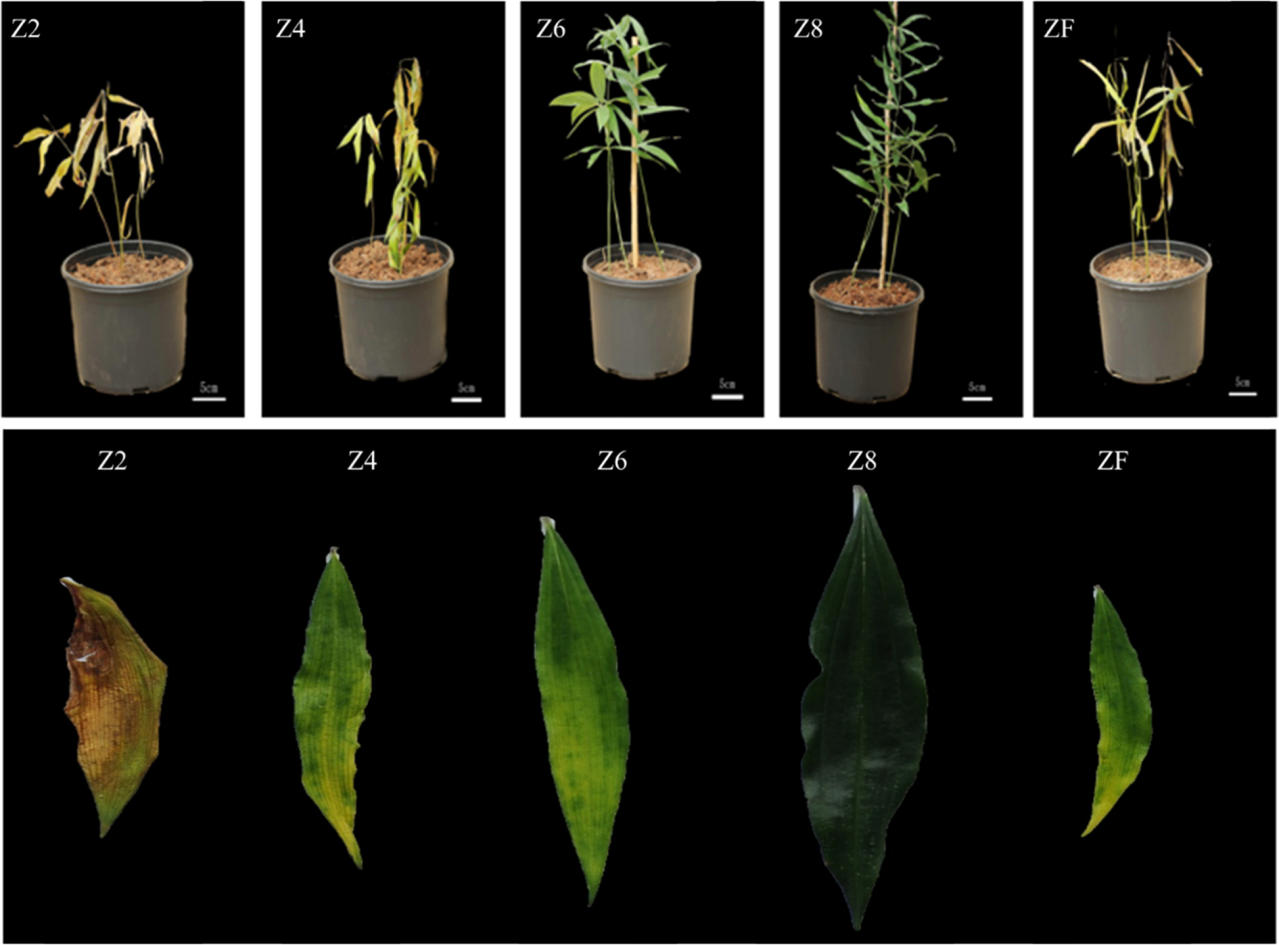
Morphological observation of *P. kingianum* plants in response to different drought stress and rewatering treatments. The above panels represent the whole plants while the lower panel represent the leaves. Control (Z8), mild drought (Z6), moderate drought (Z4), severe drought (Z2), and rewatered samples (ZF)

**Fig. 2:**
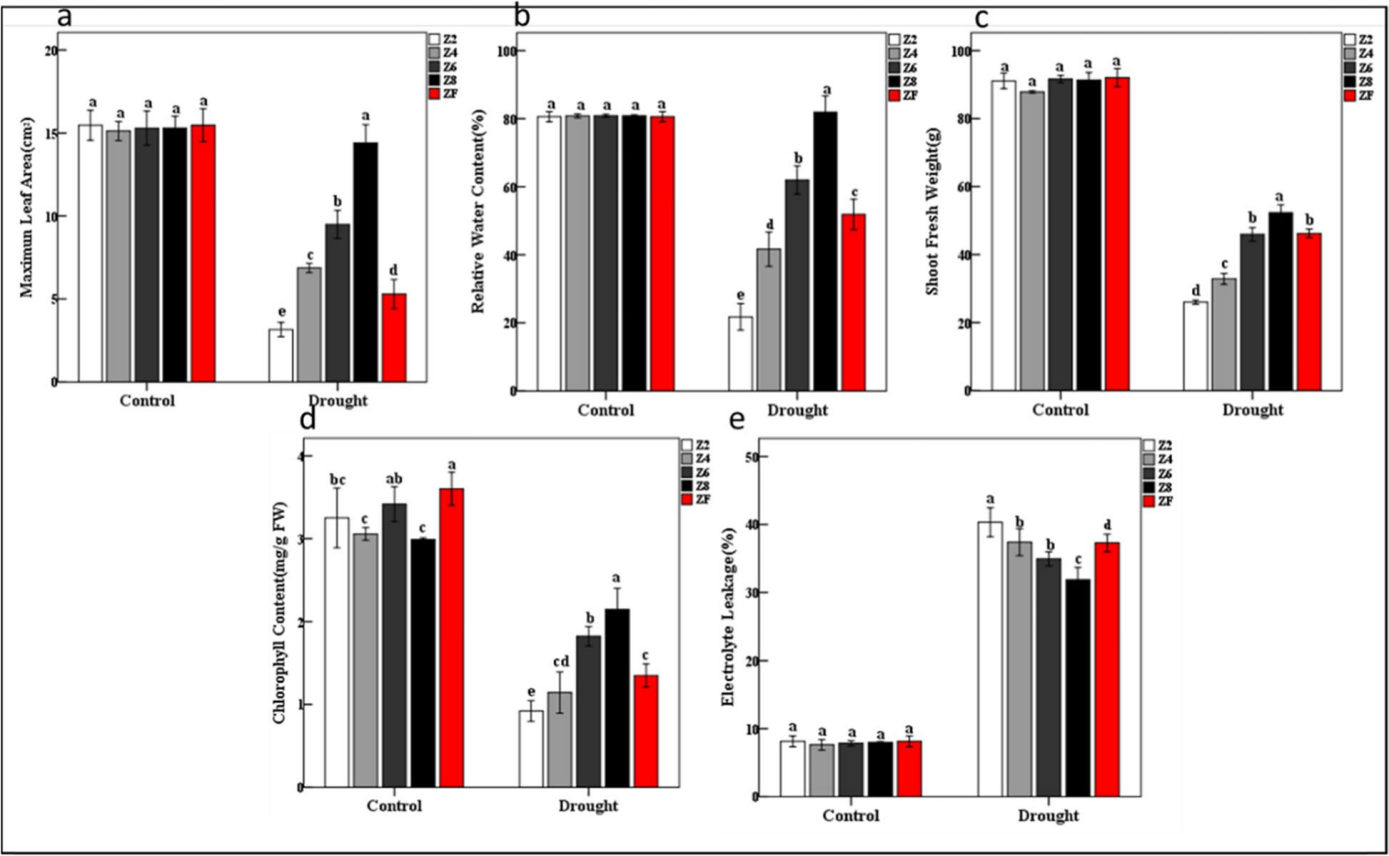
Physiological response of *P. kingianum* plants to drought and rewatering. **a** Leaf area, **b** relative water content, **c** shoot fresh weight, **d** chlorophyll content, and **e** electrolyte leakage. Control (Z8), mild drought (Z6), moderate drought (Z4), severe drought (Z2), and rewatered samples (ZF)

Our results demonstrated that the plants challenged with the different levels of drought responded differently to the applied stress. The plants showed successive drying, wilting, and yellowing of the leaves from Z6, Z4, to Z2 as compared to Z8. The rewatered plants (ZF) showed slight recovery (Fig. 1). The leaf area was significantly affected by drought treatments. The maximum decrease in the leaf area was observed for Z2 followed by Z4, and Z6 as compared to Z8. The rewatering slightly increased the leaf area as compared to ZF (Fig. 2a). The drought affected the relative water content (Fig. 2b), shoot fresh weight (Fig. 2c), and chlorophyll content (Fig. 2d) in a similar way i.e., the reduction in these traits was dependent on the intensity of the applied drought. Whereas the ZF plants showed a slight improvement as compared to sever and moderate drought. The electrolyte leakage was maximum in case of Z2 and minimum for the plants Z6 plants (Fig. 2e). Together, these observations propose that the *P. kingianum* plants are differently affected by the intensity of drought i.e., the effects were severe to less sever for Z2 to Z6 plants.

### *P. kingianum* tuber transcriptome profile under drought stress

**Fig. 3:**
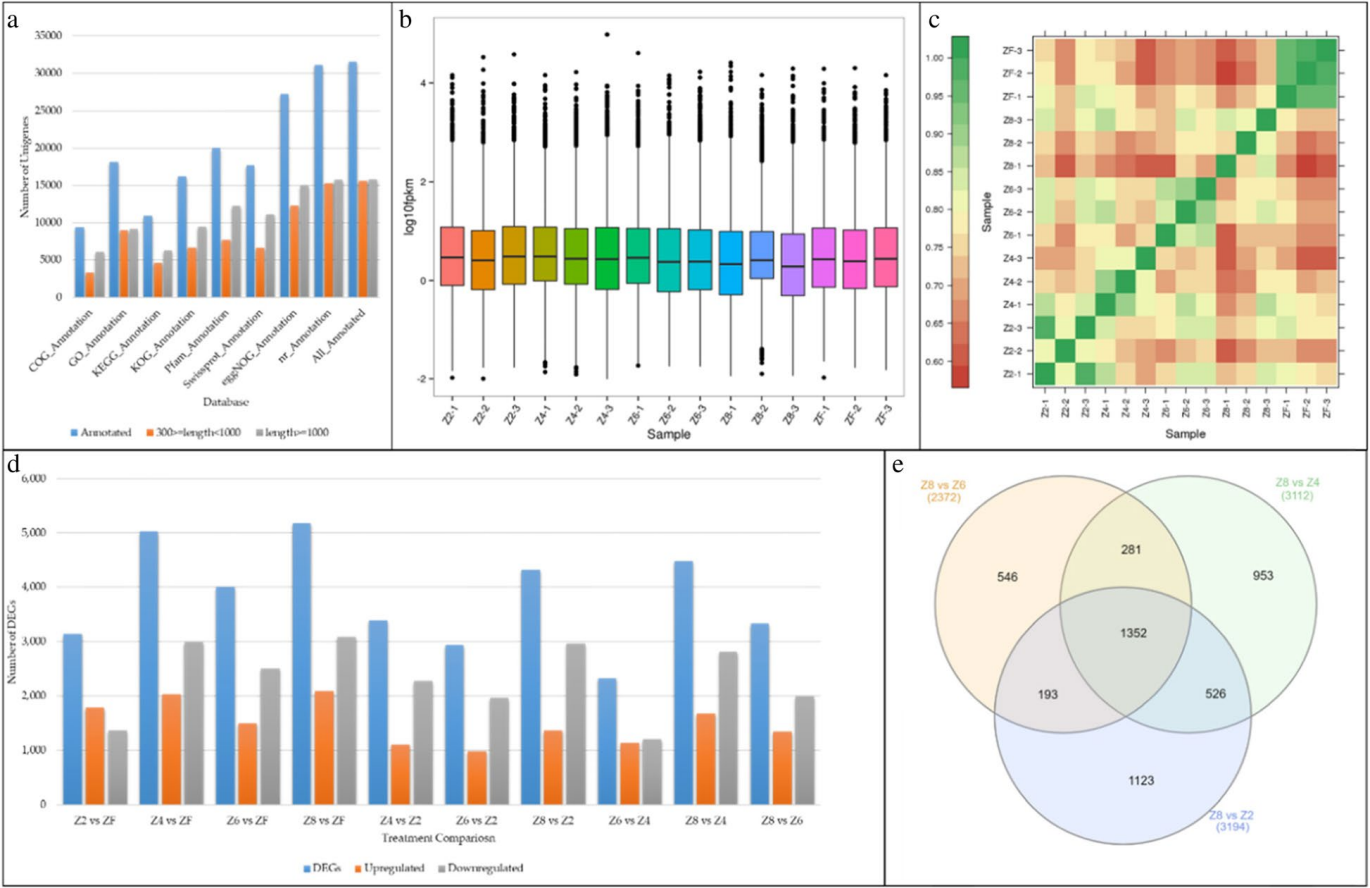
Summary of RNA-sequencing. **a** Statistics of unigene annotation to different databases, **b** overall distribution of *P. kingianum* gene expression, **c** Pearson correlation between treatments (as well as between replicates), **d** statistics of differentially expressed genes in different treatment comparisons, and **e** Venn diagrams showing common differentially expressed genes between drought treatments in *P. kingianum*. Control (Z8), mild drought (Z6), moderate drought (Z4), severe drought (Z2), and rewatered samples (ZF)

The transcriptome sequencing of 15 samples belonging to four treatments and control resulted in a total of 98.89 Gb clean data (5.99 Gb/sample with Q30 base percent of 93.74%). The summary of the *P. kingianum* tuber transcriptome statistics is presented in Table 1. After assembly 53,081 unigenes were obtained including 20,281 unigenes with a length of more than 1 kb. More than 59% of the unigenes (31,466) could be annotated to different databases (Fig. 3a). Overall distribution of sample gene expression i.e., Fragments Per Kilobase of Transcript per Million fragments mapped (FPKM) is shown in Fig. 3b. A stronger correlation was observed between samples (Fig. 3c), indicating that the sampling was reliable.

**Table 1:** Transcriptome sequencing statics summary

Sample-ID	Read Number	Base Number	GC Content %	≥Q30	Clean Reads	Mapped Reads	Mapped Ratio
Z2–1	21,123,676	6,306,330,094	48.07%	94.35%	21,123,676	15,523,081	73.49%
Z2–2	21,397,167	6,397,690,296	47.66%	94.35%	21,397,167	16,144,576	75.45%
Z2–3	22,236,972	6,637,009,552	48.08%	94.35%	22,236,972	15,985,306	71.89%
Z4–1	21,118,593	6,305,106,924	46.43%	94.17%	21,118,593	14,806,296	70.11%
Z4–2	23,790,614	7,090,266,888	47.28%	94.57%	23,790,614	16,560,974	69.61%
Z4–3	23,956,150	7,135,358,228	48.42%	94.23%	23,956,150	16,806,382	70.15%
Z6–1	21,575,434	6,435,443,806	47.93%	94.63%	21,575,434	15,028,636	69.66%
Z6–2	24,131,601	7,209,023,198	47.97%	94.42%	24,131,601	17,463,108	72.37%
Z6–3	22,982,226	6,864,034,146	48.00%	94.62%	22,982,226	16,443,549	71.55%
Z8–1	22,550,026	6,730,389,180	47.74%	94.03%	22,550,026	16,839,244	74.68%
Z8–2	22,140,956	6,606,765,896	48.81%	94.37%	22,140,956	15,520,867	70.10%
Z8–3	22,952,467	6,856,879,424	47.89%	94.69%	22,952,467	16,931,770	73.77%
ZF-1	20,961,352	6,265,824,700	48.30%	93.74%	20,961,352	15,390,355	73.42%
ZF-2	20,063,367	5,988,335,510	47.46%	94.16%	20,063,367	14,839,114	73.96%
ZF-3	20,325,546	6,063,480,826	47.63%	94.20%	20,325,546	14,946,880	73.54%

#### Differential gene expression of P. kingianum tuber under drought stress

**Fig. 4:**
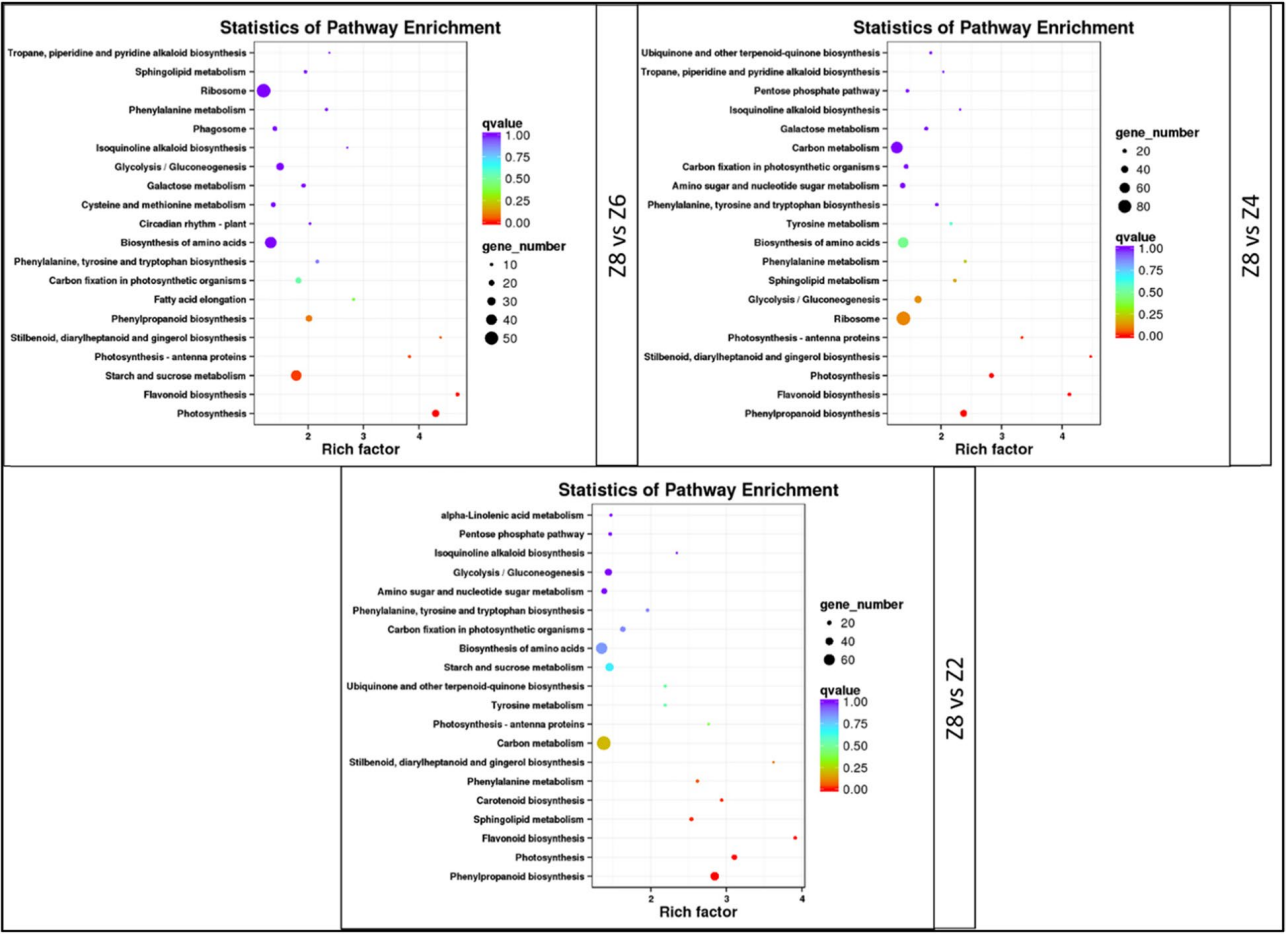
KEGG pathway (https://www.kegg.jp/kegg/kegg1.html) enrichment with differentially expressed genes between different drought treated *P. kingianum* and rewatered tubers. The soil water content was Z8 (80%), Z6 (60%), Z4 (40%), and Z2 (20%) of the maximum water holding capacity of the field soil

The control treatment Z8 (80% soil water content) had the highest number of differentially expressed genes (DEGs) (5176) as compared to the drought treatments Z4 (5,023), Z6 (4,002), and Z2 (3,147) (Fig. 3d; Supplementary Tables S1, S2, S3). Of these, 1352 DEGs were common in the three drought treatments as compared to control (Z8) (Fig. 3e; Supplementary Table S4). Almost half of these DEGs (697) were exclusively expressed in Z8 with no expression in either of the drought treated *P. kingianum* tubers. Top significantly enriched pathways between Z8 and Z6 were photosynhthesis, flavonoid biosynthesis, starch and sucrose metabolism, photosynthesis-antenna proteins, stilbenoid, diarylheptanoid and gingerol biosynthesis, and phenylpropanoid biosynthesis. Genes that were differentially expressed between Z8 and Z4 were enriched in the same pathways except for starch and sucrose metabolism. Under extreme drought i.e. Z2, we noticed the enrichment of DEGs in phenylpropanoid biosynthesis, photosynthesis, flavonoid biosynthesis, sphingolipid metabolism, carotenoid biosynthesis, phenylalanine metabolism, and stilbenoid, diarylheptanoid and gingerol biosynthesis pathways (Fig. 4). These results suggest that sphingolipid metabolism, carotenoid biosynthesis, and phenylalanine metabolism pathways are affected when the stress level is maximum i.e. Z2 in our study. The changes in the expression of key genes propose that the regulation of photosynthesis-antenna proteins and starch and sucrose metabolism are specific to both mild (Z4) and moderate (Z6) drought stresses.

#### Differential gene expression of P. kingianum tuber in recovery from drought damage

**Fig. 5:**
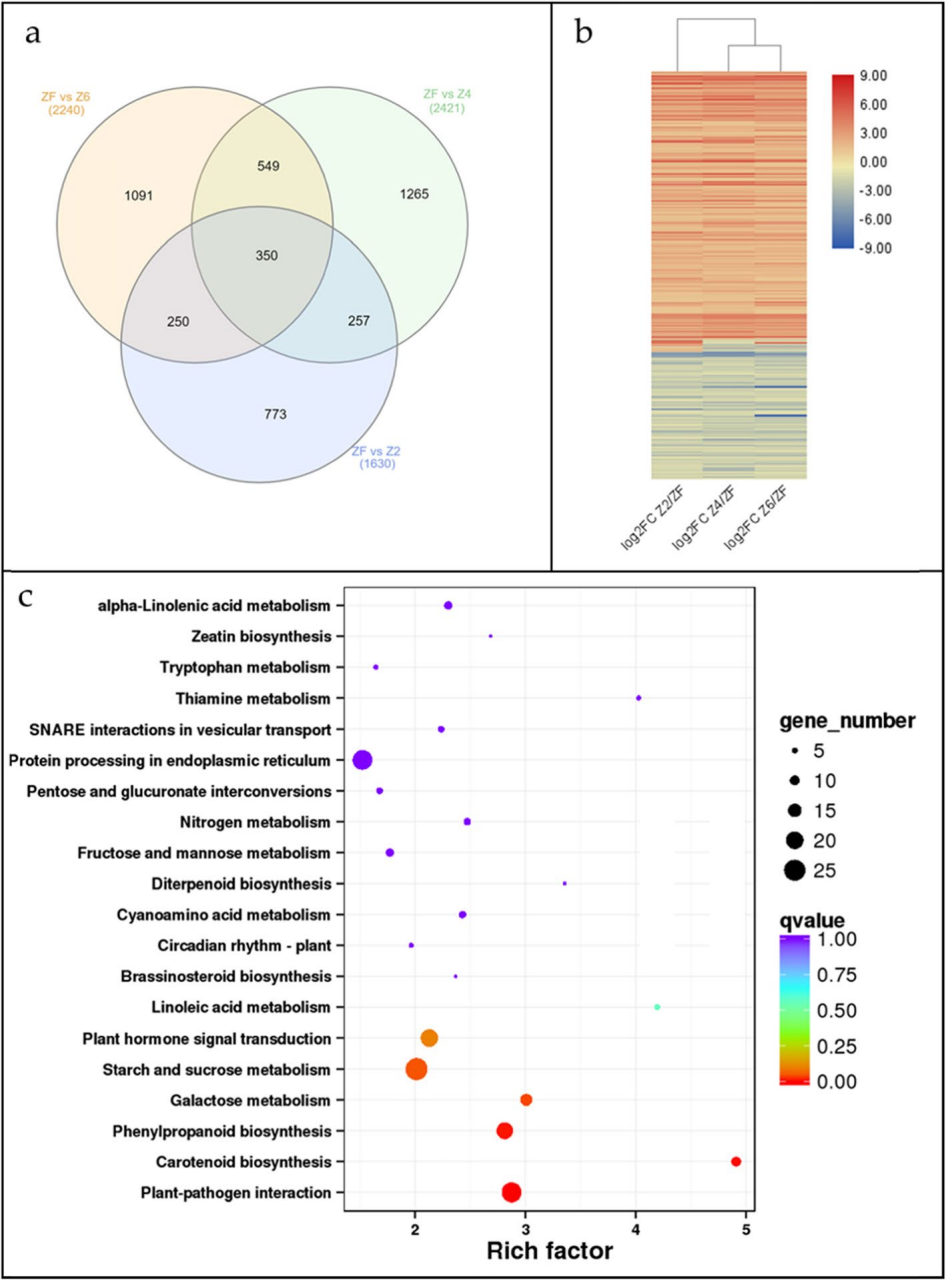
Differential gene expression of *P. kingianum* tuber in recovery from drought. **a** Venn diagram representing common DEGs expressed between drought and rewatering treatments, **b** Heatmap of log2FC values of common DEGs between drought and rewatering treatments, and **c** scatter chart displaying KEGG pathway (https://www.kegg.jp/kegg/kegg1.html) enriched between ZF and Z2. Control (Z8), mild drought (Z6), moderate drought (Z4), severe drought (Z2), and rewatered samples (ZF)

A higher number of genes (5023) were differentially expressed between drought treatment Z4 and rewatering (ZF) followed by 4002 and 3147 DEGs in Z6vsZF and Z2vsZF, respectively (Fig. 3d). We found that 350 core DEGs (set of DEGs that were expressed in all treatment comparisons) were common in comparisons of drought treatments i.e. Z6, Z4, and Z2 with ZF (Fig. 5a). Of these, 229 DEGs were upregulated, 109 were downregulated in ZF as compared to drought treatments, and 12 were regulated differently in different treatment comparisons (Fig. 5b). Apart from these comparisons, we specifically focused on the comparison between the extreme stress and rewatering treatments i.e. Z2vsZF. Out of 3147 DEGs, 133 and 21 were exclusively expressed in ZF and Z2, respectively (Supplementary Table S5). The DEGs expressed between Z2 and ZF were enriched in plant-pathogen interaction, carotenoid biosynthesis, phenylpropanoid biosynthesis, galactose metabolism, and starch and sucrose metabolism (Fig. 5c). When we compared these pathways with the drought vs control treatment comparisons, it could be stated that galactose metabolism and plant-pathogen interaction pathways are specifically regulated under rewatering conditions, while other pathways were commonly regulated between drought and rewatering in *P. kingianum* tuber.

#### Regulation of secondary metabolites related pathways

**Fig. 6:**
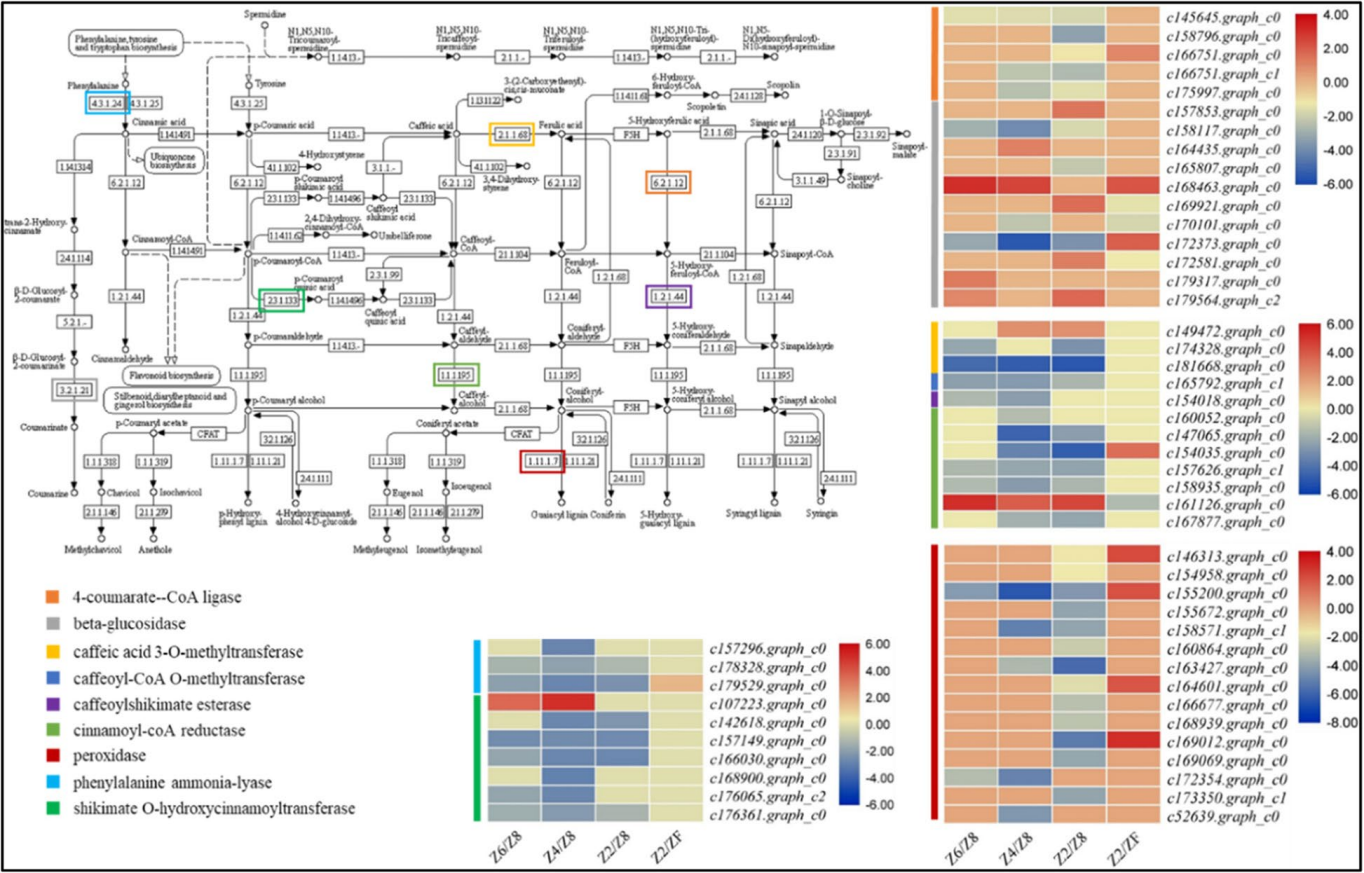
Differential regulation of phenylpropanoid biosynthesis pathway in *P. kingianum* tuber in drought and rewatering conditions. The heatmaps show log2FC values of the DEGs. The color of the bars and boxes on the pathway represent the gene(s). Control (Z8), mild drought (Z6), moderate drought (Z4), severe drought (Z2), and rewatered samples (ZF). The pathway was obtained from the KEGG database (https://www.kegg.jp/kegg/kegg1.html)

Drought stress causes oxidative stress in plants and alters the biosynthesis of flavonoids, phenylpropanoids, and, other secondary metabolites [29]. The KEGG pathway analysis showed the enrichment of DEGs in secondary metabolite biosynthesis pathways. A relatively higher number of DEGs were enriched in the phenylpropanoid biosynthesis pathway. Three phenylalanine ammonia-lyase (PALs) and four 4-coumarate-coA ligases (4CLs) were downregulated either in one or more drought treatment comparisons with the control. Bet-glucosidases had variable expression pattern i.e., some were upregulated while others were downregulated in drought treated tubers. Similar expression pattern was noticed for caffeic acid 3-O-methyltransferases (COMT), while caffeoyl-CoA O-methyltransferase (*c165792.graph_c1*), cinnamoyl-coA reductase (CCR, *c160052.graph_c0*), and caeffeoylshikimate esterase (CSE, *c154018.graph_c0*) were downregulated in drought treated tubers. The cinnamyl-alcohol dehydrogenases were downregulated in drought stress except for one gene (*c161126.graph_c0*, upregulated in drought). Suggesting differential regulation of the same genes under the same conditions. Fifteen peroxidases (PODs) were downregulated in at least one drought treatment. Many other genes (*c169832.graph_c0*; coumaroylquinate (coumaroylshikimate) 3′-monooxygenase, *c166621.graph_c0*; ferulate-5-hydroxylase (FAH), *c154189.graph_c0*; norbelladine O-methyltransferase, *c144288.graph_c0*; peroxiredoxin 6, 1-Cys peroxiredoxin, and *c167521.graph_c0*; trans-cinnamate 4-monooxygenase), and shikimate O-hydroxycinnamoyltransferases (HSTs) also showed downregulation in drought treatment (Fig. 6).

The expression of a 4CL, four PODs, ferulate-5-hydroxylase, and PAL was altered (upregulated) after rewatering. Suggesting their function returned to normal in ZF. Beta-glucosidases and cinnamyl-alcohol dehydrogenases showed variable expression in rewatered tubers similar to drought stresses. These observations clearly indicate that overall drought stress has negative effect of the phenylpropanoid biosynthesis pathway, and rewatering recovers the expression of many genes in this pathway (Fig. 6).

**Fig. 7:**
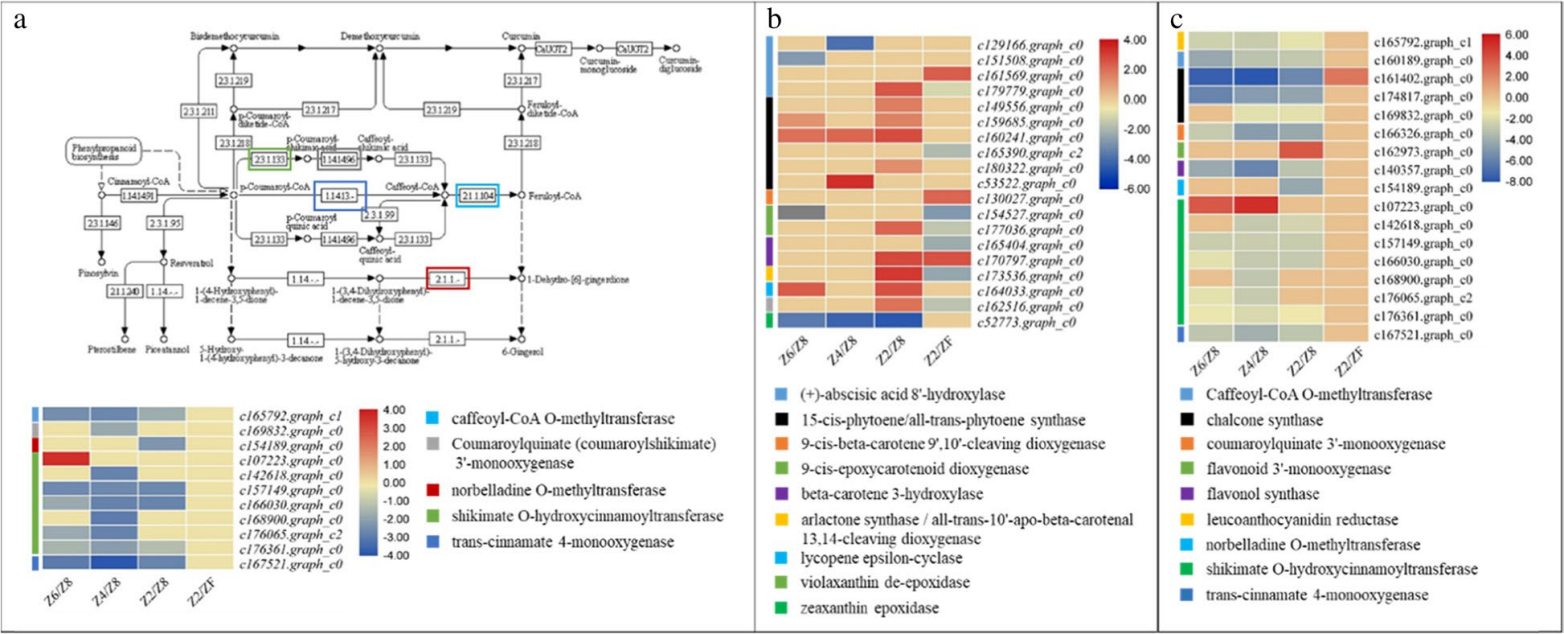
Differential regulation of **a** stilbenoid, diarylheptanoid and gingerol biosynthesis pathway, **b** carotenoid biosynthesis pathway, and **c** flavonoid biosynthesis pathway in *P. kingianum* tuber in drought and rewatering conditions. The heatmaps show log2FC values of the DEGs. The color of the bars and boxes on the pathway represent the gene(s). Control (Z8), mild drought (Z6), moderate drought (Z4), severe drought (Z2), and rewatered samples (ZF). The pathway was obtained from the KEGG database (https://www.kegg.jp/kegg/kegg1.html)

Eleven DEGs enriched in stilbenoid, diarylhepatnoid and gingerol biosynthesis pathway were regulated by drought stress. All the genes were downregulated except one shikimate O-hydroxycinnamoyltransferase (*c107223.graph_c0*), which only showed upregulation in Z6. None of the genes were differentially regulated between Z2 and ZF. These expression patterns suggest that although drought significantly affected this pathway, rewatering had a limited role in the modification of the expression of these genes (Fig. 7a).

Nineteen DEGs were enriched in the carotenoid biosynthesis pathway. Three of four (+)-abscisic acid 8′-hydroxylases were downregulated in drought indicating the decreased production of phasic acid in drought stress. One (+)-abscisic acid 8′-hydroxylase was upregulated and one was downregulated in the rewatered tuber. Six 15-cis-phytoene/all-trans-phytoene synthases (PSYs) were upregulated in at least one drought treatment. A 15-cis-phytoene/all-trans-phytoene synthase (*c165390.graph_c2*) was downregulated in rewatered tuber. These genes convert generyl-generyl-pp into pre-phytoene-pp, which is then converted to phytoene [30]. Therefore, it is possible that the upregulation of these genes under the influence of drought resulted in increased phytoene production. Contrarily, its production was stopped by downregulation of gene(s) in rewatered tubers. The genes that were annotated as 9-cis-epoxycarotenoid dioxygenase, carlactone synthase/all-trans-10′-apo-beta-carotenal 13,14-cleaving dioxygenase, lycopene epsilon-cyclase, and violaxanthin de-epoxidase were upregulated in drought stress and upregulated in rewatered plants. Zeaxanthin epoxidase was downregulated in all drought treatments while no differential regulation was observed in rewatered plants (Fig. 7b).

All the genes in the flavonoid biosynthesis pathway were downregulated in drought treated tubers as compared to ZF except one shikimate O-hydroxycinnamoyltransferase gene (*c107223.graph_c0*). Another notable observation was the rewatering driven upregulation of a chalcone synthase (*161,402.graph_c0*) (Fig. 7c).

#### Regulation of galactose metabolism and starch and sucrose metabolism pathways

**Fig. 8:**
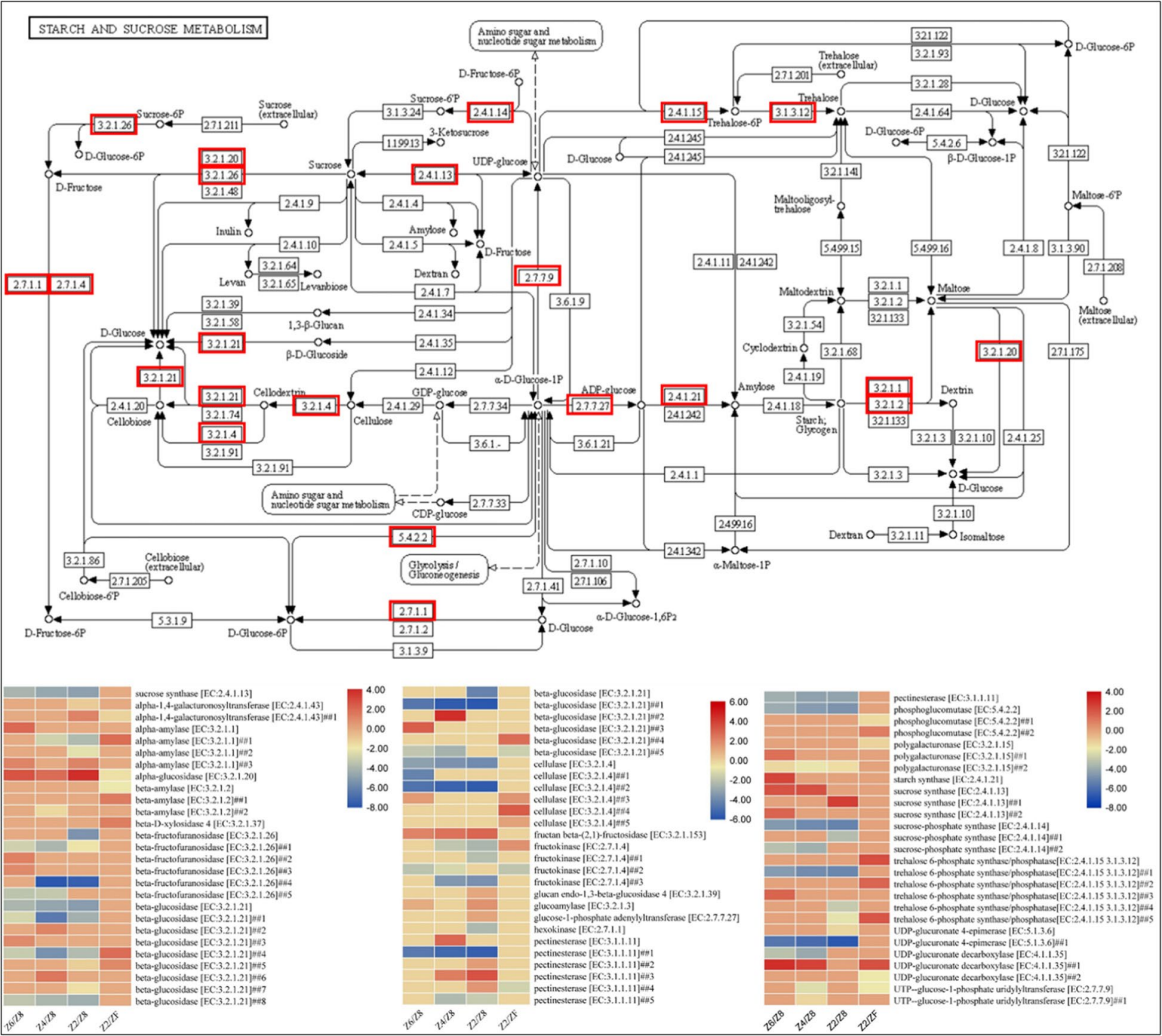
Heatmaps of log2FC values of DEGs that were enriched in starch and sucrose metabolism pathway in *P. kingianum* tuber in drought and rewatering conditions. The red boxes on the pathway show the reactions steps being differentially regulated. The E.C. numbers on the heatmaps correspond to the ones in the pathway. Control (Z8), mild drought (Z6), moderate drought (Z4), severe drought (Z2), and rewatered samples (ZF). The pathway was obtained from the KEGG database (https://www.kegg.jp/kegg/kegg1.html)

Eighty-one DEGs were enriched in galactose metabolism as well as starch and sucrose metabolism in *P. kingianum* tubers grown under drought and rewatering conditions (Fig. 8). One sucrose synthase was downregulated and three were upregulated in drought treated tubers suggesting increased sucrose synthesis in drought stress. Similarly, starch synthase was also upregulated in drought stress. The enzymes sucrose-phosphate synthases, which catalyzes the rate-limiting step in sucrose synthesis [31], were downregulated. The enzymes trehalsoe-6-phosphate synthases that break down UDP-glucose into trehalose were downregulated in drought and upregulated in rewatering. We noticed the upregulation of alpha-glucosidase in drought and downregulation in rewatered plants. This enzyme converts sucrose into D-fructose suggesting that the available sucrose is being converted and used for plant survival. Fructokinase that further breaks down the D-fructose was downregulated. Beta-amylases and 6 betafructofuranosidases showed varied expressions. Most beta-glucosidases were downregulated in drought and upregulated in rewatering. Upstream of these genes, cellulases are present which showed a similar expression pattern indicating a reduced synthesis of D-glucose in drought and a normal production in the recovery process.

These expression changes suggest that sucrose production increased in drought stress and its conversion into D-fructose was increased. The UDP-glucose break down was reduced in drought stress and increased in rewatered plants.

#### Regulation of plant-pathogen interaction pathway

**Fig. 9:**
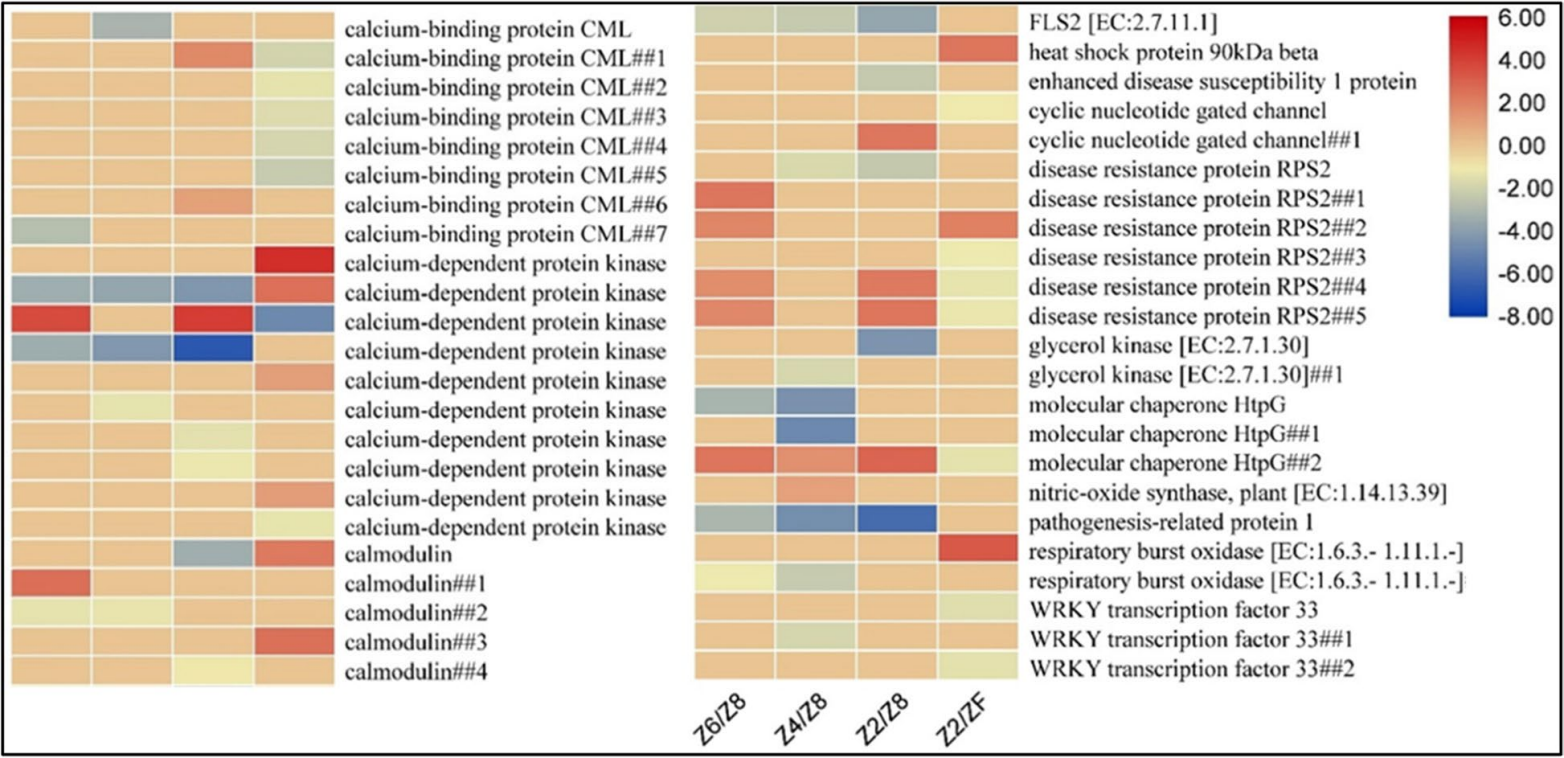
Heatmaps of log2FC values of DEGs that were enriched in plant-pathogen interaction pathway in *P. kingianum* tuber in drought and rewatering conditions. Control (Z8), mild drought (Z6), moderate drought (Z4), severe drought (Z2), and rewatered samples (ZF)

Forty-six DEGs were enriched in the plant-pathogen interaction pathway. Two of eight calcium-binding protein CMLs were upregulated in Z2 as compared to Z8. One CML was downregulated in Z6, while another was downregulated in Z4. After rewatering five CMLs downregulated. Of the 10 calcium-dependent protein kinases (CDPKs), only one (*c149342.graph_c0*) was upregulated in drought and downregulated in rewatered tubers, while others were downregulated in at least one drought treatment and upregulated in rewatered tubers. A similar expression pattern was observed for calmodulin gene family members where most showed downregulation in drought and upregulation in rewatered plants, while only one gene (*c149788.graph_c0*) was upregulated in mild drought stress. Three WRKY33s were expressed; one was downregulated in Z4 and two were downregulated in ZF. A flagellin sensing 2 (FLS2) gene was downregulated in all drought treatments. A heatshock protein coding gene was upregulated (*c175581.graph_c0*) in rewatered plants. Two cyclic nucleotide-gated channels also differentially expressed in Z2 and ZF. Six members of RPS2 (disease resistance protein) were also differentially expressed in at least one drought treatment or rewatered plants. The expression pattern of these genes was variable. Glycerol kinases were downregulated in drought. One respiratory burst oxidase (Rboh) was upregulated in ZF and one was downregulated in Z2 and Z4 (Fig. 9).

#### Differential expression of transcription factors in P. kingianum tubers in response to drought and rewatering

**Fig. 10:**
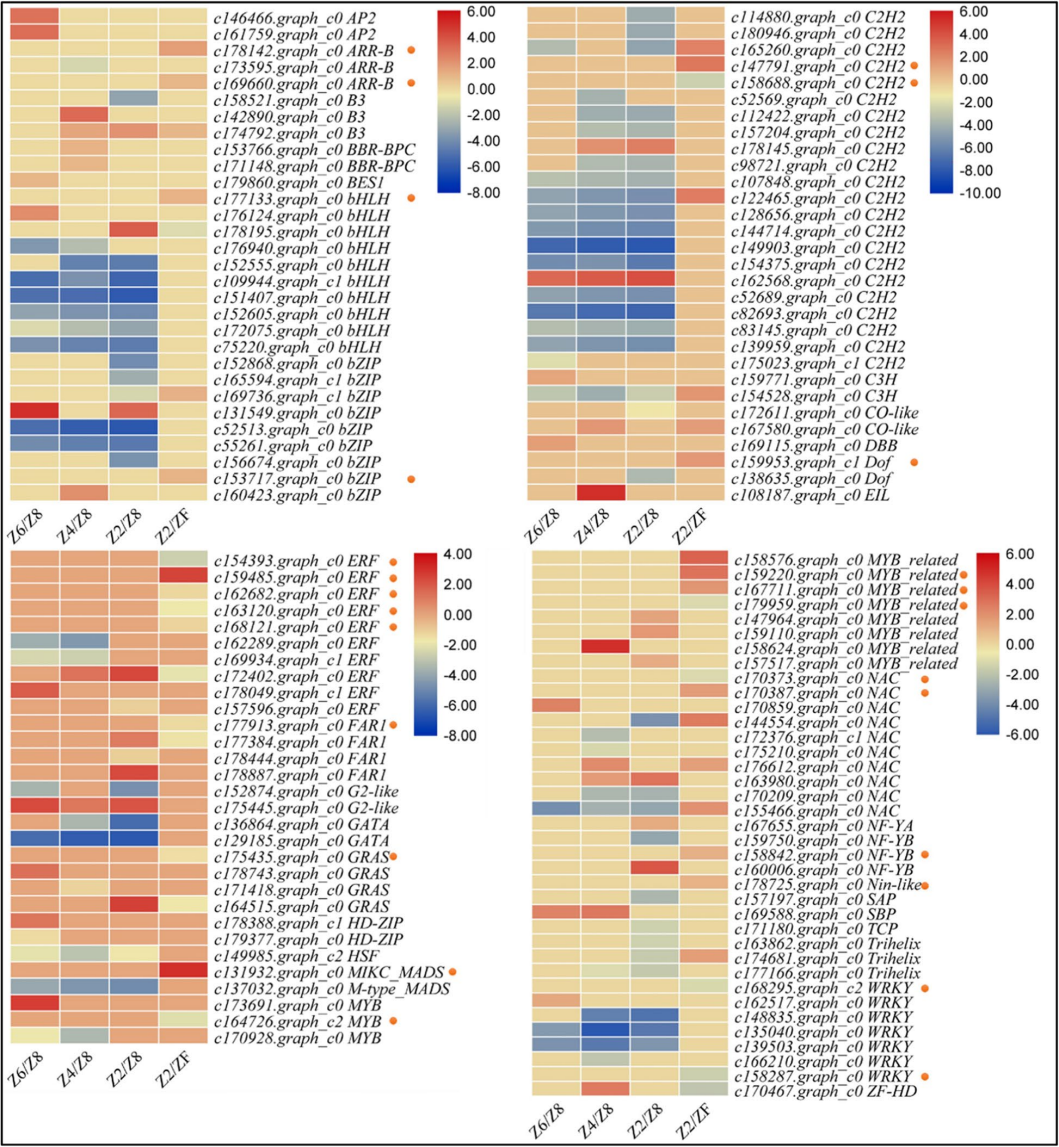
Heatmaps representing log2 foldchange values of transcription factors that were differentially expressed between different treatments of drought treated as well as rewatered *P. kingianum* tubers. The transcription factors highlighted with an orange dot are solely expressed between Z2 and ZF. Where, Control (Z8), mild drought (Z6), moderate drought (Z4), severe drought (Z2), and rewatered samples (ZF)

The transcriptome sequencing showed the expression of 458 transcription factors (TFs) of which only 127 were differentially expressed in the four comparisons i.e., Z8 vs Z2, Z8 vs Z4, Z8 vs Z6, and Z2 vs Z8. These TFs belong to 33 different families (Fig. 10). In particular, we observed that most of the bHLH TFs were downregulated in drought treated tuber as compared to Z8. Interestingly, these TFs were not differentially regulated between Z2 and Z8 except one (*c177133.graph_c0*). Similar expression trend was observed for Bzip and C2H2 TFs. Two AP2 (APTELA2, *c146466.graph_c0* and *c161759.graph_c0*), BES1 (BRI1-Ethylmethylsulfone-Suppressor, *1 c179860.graph_c0*), DBB (double-box Zinc finger, *c169115.graph_c0*), ERF (*c178049.graph_c1*), GRAS (*c178743.graph_c0*), HD-ZIP (*c178388.graph_c1*), MYB (*c173691.graph_c0*), NAC (*c170859.graph_c0*), and a WRKY (*c162517.graph_c0*) were specifically upregulated in Z6 as compared to Z8. Overall, we observed that 26 TFs were differentially expressed between the drought treated tubers as compared to Z8. Of these, two were upregulated in drought conditions as compared to Z8 (*c175445.graph_c0* and *c162568.graph_c0*), while rest of the 24 TFs were downregulated in drought treated tubers. These changes suggest that drought severely affect the expression of bHLH, bZIP, C2H2, C3H, GATA, HSF, M-type MADs, and WRKYs. Other than these, we observed that twenty-six TFs were specifically differentially expressed between severe drought treated and rewatered plants (see gene IDs with * in Fig. 10). These TFs belonged to 15 different families indicating that a large number of TFs play role in transcriptional reprogramming when *P. kingianum* plants are rewatered after severe drought stress. Particularly, the increased expression of the 14 TFs in ZF as compared to Z2 shows their involvement in the repair mechanisms after rewatering in *P. kingianum* tubers.

#### qRT-PCR gene expression analysis

**Fig. 11:**
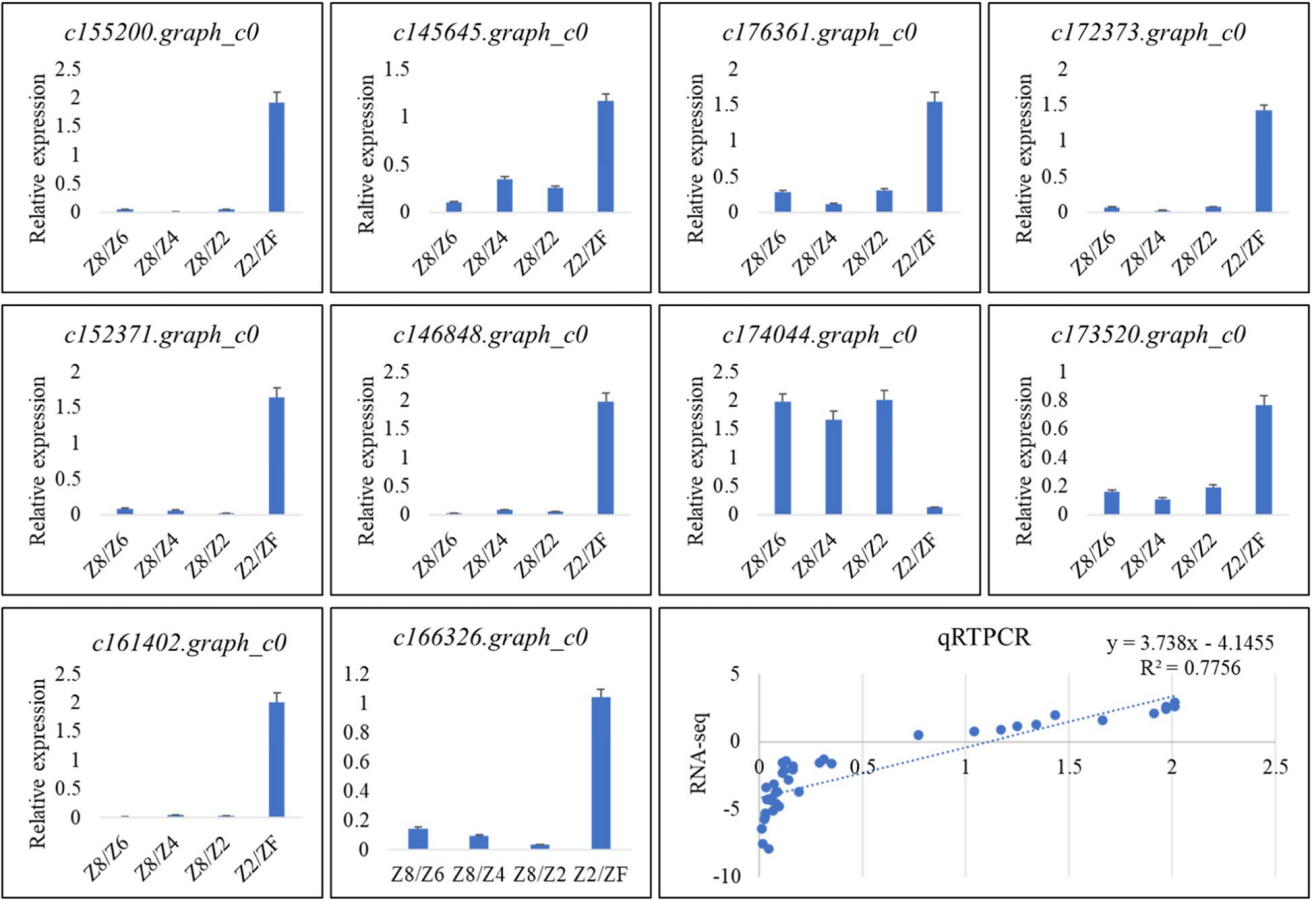
Relative gene expression of ten *P. kingianum* genes and the correlation between RNA-seq and relative gene expression

We validated the reliability of the transcriptome sequencing by determining the expression of ten randomly selected genes (Fig. 11). The genes’ expression showed a positive correlation with the FPKM values (R^2^ = 0.77). Overall, the qRT-PCR expression followed the expression pattern of the transcripts, thus confirming the reliability of the transcriptome sequencing.

## Discussion

### Drought stress severely effects P. kingianum plants

Drought stress is an inevitable factor that hampers plant biomass production, quality, and quantity (yield/energy). It is directly associated with temperature, light, and rainfall dynamics in an ecosystem [32]. Limited work has been reported on the effect of drought on *P. kingianum* plants and specifically on the tubers. A recent study reported that drought might effect rhizome bud dormancy [33] but the information on the overall plant growth is scarce. Our results clearly indicate that the severity of drought significantly affects *P. kingianum* plants as visible from the reduction in leaf area, relative water content, shoot fresh area, and chlorophyll contents (Fig. 2). These changes are in accordance with the earlier reports in different plants. For example, it is known that drought reduces cotton leaf area by 30% [34]. Similarly, in wheat it was reported that relative water content and chlorophyll content were directly linked with drought stress [19]. Decline in shoot fresh weight is a known phenomenon in different plant species e.g., citrus [35]. Thus, our observation in drought affected *P. kingianum* also suggests that similar mechanism exists in this species. The increase in osmolyte leakage with increase in drought intensity indicates that higher membrane damage was caused to *P. kingianum* leaves [36]. The overall increase in relative water content, shoot fresh weight, and chlorophyll content, and decrease in electrolyte leakage in *P. kingianum* plants is indicative of the fact that rewatering helps the plants to recover from drought effects to some extent [37]. Based on these observations, it could be concluded that drought stress significantly hampers growth and development of *P. kingianum* plants and rewatering after severe drought could help the plants in recovery to some extent.

### Lignin biosynthesis pathway in P. kingianum tubers is affected by drought stress and recovered by rewatering

Lignin has an important role in the plant’s defense against abiotic stresses as it reduces transpiration and helps to maintain the osmotic balance and protects the integrity of membranes [38]. Our results propose that drought stress strongly affected the biosynthesis of lignin as evident by the downregulation of many key enzymes in the phenylpropanoid biosynthesis pathway in drought-affected *P. kingianum* tubers (Fig. 6). Firstly, the conversion of phenylalanine into cinnamic acid was reduced due to the downregulation of PALs. Following this, the downregulation of genes in downstream the pathway such as the conversion of caffeic acid to ferulic acid by COMTs, conversion of 5-hydroxyferrulic acid into 5-hydroxy-feruloyl-CoA by 4CLs and subsequently to 5-hydroxyconiferaldehyde by CSE indicates drought affects the biosynthesis of lignin. These observations are in agreement with the changes in the expression of lignification related enzymes under water deficit. For example, it was reported that the expression of a maize COMT decreased in water deficit conditions and lignin accumulation decreased [39]. Similarly, the overexpression of 4CL in *Fraxinus mandschurica* increased osmotic stress tolerance [40]. In addition to the genes controlling above mentioned enzymes, the biosynthesis of p-coumaroyl quinic acid by HSTs and production of caffeoyl-alcohol from caffeyl-aldehyde by CCRs was also possibly affected under the effect of drought stress (as evident from the changed expression of genes in this pathway). All these reactions take part in lignin biosynthesis and hence it could be stated that the changes in the expression of these key genes significantly alters the process of lignin biosynthesis [41–43]. We say this because we also noticed the downregulation of fifteen PODs [44]. On the contrary, the upregulation of PAL, FAH, PODs, and 4CLs after rewatering the drought treated *P. kingianum* dictates that under normal irrigated conditions, the production of polysaccharides is returned to normal (Fig. 5) [45]. Hence, the genes that recovered their expression after rewatering are prime targets for genetic engineering *P. kingianum* to survive drought stress.

### Drought stress reduces the expression of genes related to gingerol and flavonoid biosynthesis in P. kingianum tubers

Stilbenoid, diarylheptanoid, and gingerol biosynthesis pathway is present downstream of the phenylpropanoid pathway and studies have shown that drought significantly affects this pathway in *Salix, Paeonia* section *Moutan* DC, and potato [46–48]. Downregulation of the enzymes catalyzing key steps such as the biosynthesis of p-coumaroyl shikimic acid, caffeoyl-shikimic acid, caffeoyl-CoA, feruloyl-CoA, and 1-dehydro-[6]-gingeroline signifies that drought has affected the production of these compounds (Fig. 4a). Overall, these steps affect the productions of 6-gingerol [49]. Thus it could be stated that drought stress downregulates the genes that are invovled in the biosynthesis of gingerol, which is consistent with previous reports that the gingerol biosynthesis pathway is regulated by abiotic stresses [50, 51]. This is an important consideration for the production of 6-gingerol owing to its importance in the health industry [52]. Another pathway that works downstream of phenylpropanoid biosynthesis pathway is the flavonoid biosynthesis pathway. The downregulation of all the genes in the flavonoid biosynthesis pathway indicates that when *P. kingianum* suffers from drought stress the production of flavonoids in the tubers is decreased (Fig. 7c). Particularly, the downregulation suggests a possible reduction in the accumulation of luteoforol, 5-deoxyleucocyanidin, caffeoyl-CoA, and feruloyl-CoA. Since most of these products are common with the gingerol biosynthesis pathway and the phenylpropanoid biosynthesis pathway, therefore, the overall effect of the drought stress possibly decreased the contents of phenylpropanoids in *P. kingianum.* Phenylpropanoids and their derivatives are also associated with estrogenic/antiestrogenic action and reduce the risk of cancer, osteoporosis, and cardiovascular diseases in humans [53, 54]. These results are important since *P. kingianum* tubers are used for the extraction of health beneficial compounds [55].

### Carotenoid biosynthesis pathway is activated in response to drought stress

Plants have evolved strategies to adapt to extreme environmental conditions such as drought. One strategy is the increased biosynthesis of isoprene, which includes carotenoids [56]. The upregulation of PSYs in extreme drought conditions (Z2) and downregulation in rewatered plants signify that in *P. kingianum* tubers, phytoene biosynthesis is increased under drought stress, which is consistent with the previous study where salt and drought treatments resulted in the upregulation of *OsPSY3* in rice roots and *IbPSY1* and *IbPSY2* in sweet potato stem [57, 58]. Similarly, the expression pattern of genes involved in lutein, Zeaxanthin, adonixanthin, and astraxanthin biosynthesis indicates the drought increased their production. Carotenoids are antioxidants and can detoxify reactive oxygen species. Furthermore, carotenoids also take part in the quenching of ^1^O_2_ and oxidation of β-carotene [59]. The regulation of carotenoids under water or drought stress is species-specific and varies based on intensity and duration of the stress. Because we observed the differential regulation of carotenoid biosynthesis-related genes in different intensities of drought stress (Z6, Z4, and Z2) and its recovery in ZF (Fig. 6c), therefore, it could be stated that in *P. kingianum* tubers, the higher carotenoid biosynthesis is a drought response which is not strictly associated with stress intensity. Similar responses were previously reported in beans, olive trees, alpine plants, and African eggplants [60–63].

### Drought stress and rewatering modulates changes in starch and sucrose biosynthesis

Plants remobilize available starch to release energy for their survival under abiotic stresses such as drought, salt, and temperature stresses [64]. This is evident from the upregulation of starch synthase and alpha-amylase in one of the drought-treated plants (Z6). The upregulation of another alpha-amylase (*c179275.graph_c0*) in other drought treatments is indicative that starch is being degraded in response to drought stress for providing energy. On the other hand, the upregulation of sucrose synthases is indicative of the increase sucrose synthesis under drought conditions. This is a common metabolic response to drought in drought-tolerant genotypes as previously noted in wheat [65]. Another strategy to inhibit water stress is the decreased expression of sucrose phosphate synthase. Potato plants adopt this strategy to inhibit the water-stress induced synthesis of sucrose in growing tubers [66]. The downregulation of sucrose phosphate synthases indicates that *P. kingianum* tubers restrict the sucrose synthesis from D-fructose-6P and instead synthesize sucrose from UDP-glucose by upregulating three of four sucrose synthases. We say this because of the restriction of UDP-glucose degradation to trehalose by the downregulation of trehalose 6-phosphate synthase/phosphatases. This further suggests that in *P. kingianum,* Suc-Tre6p nexus model exists and regulates sucrose levels in tuber [67], which is normalized after rewatering as evident from the upregulation of trehalose 6-phosphate synthases (Fig. 8). Further specific characterization of key players in this model in *P. kingianum* plants would shed detailed light on the signals and negative feedback regulating agents in this process. Further, it might be the case that the breakdown of cellulose and subsequent biosynthesis of D-glucose was restricted in drought treated plants and returned to normal after rewatering. We suggest this based on the changes in the expression of cellulases and beta-glucosidases and their respective positions within the pathway (Fig. 8). This could mean that *P. kingianum* does not exploit cellulose for the generation of D-glucose. However, specific gene characterization studies will reveal the details on this proposition. Overall, the changes in the expression of key genes in the starch and sucrose biosynthesis pathway indicate that drought suppresses the conversion of UDP-glucose to D-glucose and trehalose and improves sucrose as well as starch biosynthesis in *P. kingianum* tubers [68]. Rewatering the drought-affected *P. kingianum* tubers recovers them by modulating the expression of these genes.

### Drought stress and rewatering regulates plant-pathogen interaction pathway

Changes in the cell wall are a common response to biotic and abiotic stresses [69–71]. CDPKs that are a part of plant-pathogen interaction, also are involved in cell wall reinforcement [72]. Both CDKPs and CMLs affects cell wall by the generation of reactive oxygen species and nitric oxide [73]. The differential expression of CDPKs and CMLs in drought stress treated and rewatered *P. kingianum* tubers is consistent with their previously established roles [74]. Another key player i.e., Rboh which is located downstream of CDPK was downregulated. The differential expression in *P. kingianum* tubers is consistent with previous work that its transcripts are abundant in underground parts (roots in Arabidopsis) [75] and establishes that in *P. kingianum* Rboh is suppressed under drought stress, due to which downstream processes such as cell wall reinforcement are affected [76].

The downregulation of FLS2 in response to drought stress in Z6, Z4, and Z2 is interesting since FLS2 is known to regulate the immune responses against biotic stresses [77]. Similarly, the disease resistance protein RPS2 showed varied expression under drought stress and rewatering. However, RPS2 is specifically known for its role in specifying the recognition of *Pseudomonas syringae* [78]. Future studies must explore the role of FLS2 and RPS2 in drought-affected plants and how it induces the expression of downstream defense-related genes in abiotic stresses. The differential regulation of heat shock protein (90kDA beta) between Z2 and ZF indicates its role in relief from drought stress after rewatering the *P. kingianum* tubers [79]. Among other key genes in the pathway, the downregulation of glycerol kinases is also indicative of drought stress response in tubers (Fig. 9). We say this because an earlier study reported that the Arabidopsis seedlings lacking glycerol kinase accumulated higher glycerol contents which proved to be better adapted to hyperosmotic and oxidative stresses [80].

### Role of transcription factors in drought stress and rewatering of P. kingianum tubers

Drought tolerance in plants is a cross-talk between certain molecular, cellular, and physiological processes that are under the control of induction/repression of various genes and transcription factors [81]. During the signal transduction, TF regulate the expression of multiple genes and act as switches due to the presence of cis-elements in their promotor region [82]. The regulation of a large number of TFs belonging to 33 families is indicative of their significant roles in drought stress tolerance and rewatering. The bHLH TFs play important roles in drought stress in addition to their roles in reproduction (flower and fruit development) [83]. The downregulation of bHLH TFs in Z2, Z4, and Z6 as compared to Z8 indicates that drought stress affects the expression of these TFs in *P. kingianum* tubers. However, their downregulation in specific to drought treatment in *P. kingianum* tubers should be further explored since earlier reports proposed that these TFs are activated in plant tissues when under stress. In this regard the upregulation of one bHLH (*c176124.graph_c0*) could propose its role in defense against the mild drought stress. Similarly, the increased expression of *c178195.graph_c0* in severe drought as compared to Z8 and downregulation in rewatered tubers is indicative of their known function as drought stress tolerance regulators [84]. This gene could be a good candidate to study and characterize in future studies and breed drought tolerant *P. kingianum* plants. Mild drought in *P. kingianum* tubers is possibly under the regulation of multiple TFs (Fig. 10). Among these, the increased expression of AP2 TFs in Z6 is quite relevant to the observations related to hormone signaling pathway. AP2 participates in multiple abiotic stress responses including drought stress responses and the activation of ABA and ethylene dependent stress-responsive genes [85]. Similarly, the increased expression of BES1, DBB, ERF, GRAS, WRKY, HD-ZIP, NAC, and MYB TFs in mild drought indicates that *P. kingianum* tubers activate a large array of networks to cope with the mild drought stress. While, in addition to these TFs, on the onset of moderate drought stress, *P. kingianum* tubers may additionally express B3, BBR-BPC, CO-like, G2-like, SBP, and ZF-HD TFs. This indicates that the severity of drought may lead to the activation of different TFs in *P. kingianum* tubers [86–88]. This was further confirmed during the severe drought that resulted in the regulation (increased expression) of FAR1, MYB-related, NF-YA, and NF-YB TFs. Earlier studies have indicated that different levels of drought stress may be regulated by manipulation of different pathways driven by the changes in the expression of TFs ([89] and references therein, [90]). There is need to understand the individual roles of the differentially expressed TFs in *P. kingianum* plants so that these might be manipulated for breeding drought stress tolerant varieties by using CRISPR/Cas and other new breeding technologies [91].

## Conclusions

The transcriptome comparison of *P. kingianum* tubers grown in mild, moderate, and severe drought, and rewatering showed that drought significantly affects lignin, gingerol, and flavonoid biosynthesis. Rewatering of the drought-affected *P. kingianum* recovers the tubers from drought effects by showing contrasting gene expression profiles. PALs, COMTs, CSE, 4CLs, HSTs, PODs, and CCRs are important target genes for higher lignin production in *P. kingianum* tubers. Phenylpropanoids biosynthesis in general and Gingerol biosynthesis in specific are negatively affected by drought stress. Carotenoids play an essential role in defense against drought stress in this species. Our transcriptome results propose that *P. kingianum* increased the biosynthesis of starch and sucrose, and Suc-Tre6p model operates in tubers to regulate sucrose levels under drought stress. Several genes such as FLS2, CMLs, CDPKs, Rboh, and RPS2 that are associated with plant-pathogen interaction are negatively regulated by drought stress. To enhance drought stress tolerance in *P. kingianum,* genes associated with plant-pathogen interaction, starch and sucrose biosynthesis, and carotenoid biosynthesis pathways are prime targets for specific characterization and genetic modification for drought tolerance. For secondary metabolite and the polysaccharide biosynthesis, genes discussed in phenylpropanoid biosynthesis pathway, starch and sucrose biosynthesis, flavonoid biosynthesis, and galactose biosynthesis should be targeted. Future studies may also elaborate how these pathways jointly work to enable *P. kingianum* tubers in specific and whole plants to cope with drought stress. In this regard, gene co-expression networks and protein-protein interaction studies will be useful together with candidate gene specific characterizations.

## Materials and methods

### Plant material and drought treatment

The red-flower line of *P. kingianum* Coll. et Hemsl. plants were grown at the research station of Yunnan University of Traditional Chinese Medicine, Kunming, China. The plant material was obtained from Yunnan University of Traditional Chinese Medicine and the formal identification of the plant material was carried out by Prof Pengzhang Ji. No voucher specimen has been deposited in a genebank and no special permission is needed to study this species. Three years old plants were used for drought stress experiment from January to December 2019. The plants were potted in 40 × 30 cm^2^ plastic pots filled with the homogenous and sterile mixture of organic fertilizer, humus soil, and sandy soil in a ratio of 2:3:5. The experiment was carried out in a greenhouse to avoid rainy days interference and the average day/night temperature and relative humidity were set 30/22 °C and 60/55%, respectively. Drought stress treatment was applied in three treatments i.e., mild drought (Z6), moderate drought (Z4), and severe drought (Z2). In addition, a control (Z8) treatment was applied for comparison. The soil water content for the four treatments was maintained at 75–80% (Z8), 55–60% (Z6), 35–40% (Z4), and 15–20% (Z2) of the maximum water holding capacity of the potted soil. All other growing conditions were kept standard. Twenty pots per treatment were maintained with one plant per pot. One pot per treatment was kept empty to estimate the amount of evaporated water from the soil surface. Every day once at dusk, the weight of each pot was measured and the moisture content was adjusted according to the treatments [92]. During October 2019, 20% of the treatment pots (4 pots per treatment) were rehydrated for 3 days to obtain rehydrated samples (ZF). Furthermore, to eliminate the influence of the weight of *P. kingianum* seedlings, a pot was destroyed after 30 and 90 days each and the fresh weight of the seedling was measured, which was used to determine the weighing standard. After completion of drought treatment, tubers were harvested from experimental plants, washed thrice with distilled water, and stored at − 80 °C for further analyses.

### Morpho-physiological analyses

Triplicate plants were used for the measurement of morpho-physiological trait evaluation. The leaf area was computed by using LI-COR 3100 leaf area meter (LI-COR Inc., Lincoln, NB) as reported earlier [16]. The relative water content and chlorophyll content were measured as reported earlier in peanut by Shivakrishna*,* et al. [93]. Shoot fresh weight was measured on an electrical weighing balance (Tecator Model 6110). Electrolyte leakage was measured as reported by Ahmadizadeh*,* et al. [94].

### RNA sequencing

#### RNA extraction, cDNA library construction, and Transcriptome sequencing

Total RNA was extracted from the tubers by using Spin Column Plant total RNA Purification Kit (Sangon Biotech, Shanghai, China). RNA purity check, quantification, and integrity check were done as described previously [95]. Further steps for cDNA library preparation and sequencing (Illumina Hiseq 2000 platform) were completed at Beijing Biomarker Biotechnology Co., Ltd., China.

#### Data analysis, assembly, and annotation

Raw data were first processed through in-house Perl scripts and clean data (clean reads) were obtained. At the same time, Q20, Q30, GC-content, and sequence duplication levels of the clean data were calculated. All the downstream analyses were based on clean data with high quality. These post-sequencing analytical procedures were done as reported earlier [96]. The transcriptome was assembled based on the two pooled files for each sample using Trinity [97].

Gene function annotation was done according to NR (NCBI non-redundant protein sequences); Pfam (Protein family); KOG/COG/eggNOG (Clusters of Orthologous Groups of proteins) [98, 99]; Swiss-Prot (A manually annotated and reviewed protein sequence database) [100]; KEGG (Kyoto Encyclopedia of Genes and Genomes) [101]; and GO (Gene Ontology) [102] databases.

#### Quantification of gene expression levels and differential analysis

The levels of gene expression were calculated by RSEM [103]. Differential expression analysis was performed using the DESeq R package (1.10.1) [104]. Genes with an adjusted *P*-value < 0.05 found by DESeq were considered as differentially expressed. The screening conditions for differential genes are |log 2 Fold Change| > = 1, and FDR < 0.05. The overall distribution of FPKM values and the PCC was computed and represented as graph and heatmap, respectively in R (www.r-project.org). We used KOBAS [105] software to test the statistical enrichment of differential expression genes in KEGG pathways [106].

#### qRT-PCR analysis

Ten *P. kingianum* genes were randomly selected to validate the RNA-sequencing results. An *Actin7* gene was used as an internal control. The PCR reactions were carried out as reported earlier [27]. The primers were designed in Primer3Plus (Table 2) [107].

**Table 2:** List of primers used for qRT-PCR analysis

Gene ID	Forward Sequences (5′-3′)	Reverse sequence (5′-3′)
*c155200.graph_c0*	GACGAAGGGTGATCT	GCGGTATTGCAGGTATAA
*c172373.graph_c0*	GAGAAGGTAGGAGG	AATGGTGTAACCTGAAGAGC
*c145645.graph_c0*	TCTGCCGACCGACAAAGA	CGAAGAAGTAGAGGTG
*c176361.graph_c0*	GGGCGGCGTAATTTATGTGC	GTAATGAGTCCGCCTTTGA
*c152371.graph_c0*	CCTGTGTCGGCCTGTATT	GCACTACTCCAAGGAACAC
*c146848.graph_c0*	CGAAGTGGAAGTAGAGGTG	GTGCAGAGTATGCTCACA
*c174044.graph_c0*	CCGAGTGGATTGGTCACC	CCAGATCGCTTGAGATGAG
*c173520.graph_c0*	GGTGGATTGAAGCGGA	CAGCAGTTGCTCGCTGAAGAT
*c161402.graph_c0*	CTGCTGAGTGCATAAGA	CCTCCCACCACCTCACTA
*c166326.graph_c0*	TTCGACGACAGCATGGA	CCAGATCGCAGAGATGAG
*Actin7*	CTCCAGAATCCTTCCAAA	GAGAAGAGGGTAGGAGG

#### Statistical analysis

Analysis of variance was performed to assess the variation among pairwise samples using the GenStat Statistical Software (version 12; VSN International, UK).

## Supplementary Information

**Additional file 1: Supplementary Table S1.** Differentially expressed genes between treatments Z6 and Z8 in *P. kingianum* tuber. **Supplementary Table S2.** Differentially expressed genes between treatments Z4 and Z8 in *P. kingianum* tuber. **Supplementary Table S3.** Differentially expressed genes between treatments Z2 and Z8 in *P. kingianum* tuber. **Supplementary Table S4.** List of core DEGs that were expressed between drought and control treatments in *P. kingianum* tuber. **Supplementary Table S5.** DEGs expressed between extreme drought (Z2) and rewatering (ZF) treatments in *P. kingianum* tuber.

**Additional file 2: Supplementary Figure S1.** KEGG pathway enrichment with differentially expressed genes between drought treated *P. kingianum* tubers. The soil water content was Z8 (80%), Z6 (60%), Z4 (40%), and Z2 (20%) of the maximum water holding capacity of the filed soil.

## Data Availability

The raw RNA-seq data has been submitted to NCBI SRA under the Project number: PRJNA691439 (https://www.ncbi.nlm.nih.gov/bioproject/?term=PRJNA691439).

## Abbreviations

DEGs: Differentially expressed genes
FPKM: Fragments Per Kilobase of Transcript per Million fragments Mapped
KEGG: Kyoto Encyclopedia of Genes and Genomes
PAL: Phenylalanine ammonia-lyase
4CL: 4-coumarate-coA ligase
COMT: Caffeic acid 3-O-methyltransferase
CCR: Cinnamoyl-coA reductase
CSE: Caeffeoylshikimate esterase
POD: Peroxidase
FAH: Ferulate-5-hydroxylase
HST: Shikimate O-hydroxycinnamoyltransferase
CML: CAM-like
CDPK: Calcium-dependent protein kinase
PSY: 15-cis-phytoene/all-trans-phytoene synthases
Rboh: Respiratory burst oxidase
