# Supplementary material for: Transcriptomic responses to drought stress in Polygonatum kingianum tuber
Source: BMC Plant Biol. 2021 Nov 15;21:537. doi: 10.1186/s12870-021-03297-8 (PMC8591914; doi:10.1186/s12870-021-03297-8)
Supplement: Supplementary file 2 — Additional file 2: Supplementary Figure S1. KEGG pathway enrichment with differentially expressed genes between drought treated P. kingianum tubers. The soil water content was Z8 (80%), Z6 (60%), Z4 (40%), and Z2 (20%) of the maximum water holding capacity of the filed soil. [file 12870_2021_3297_MOESM2_ESM.docx]

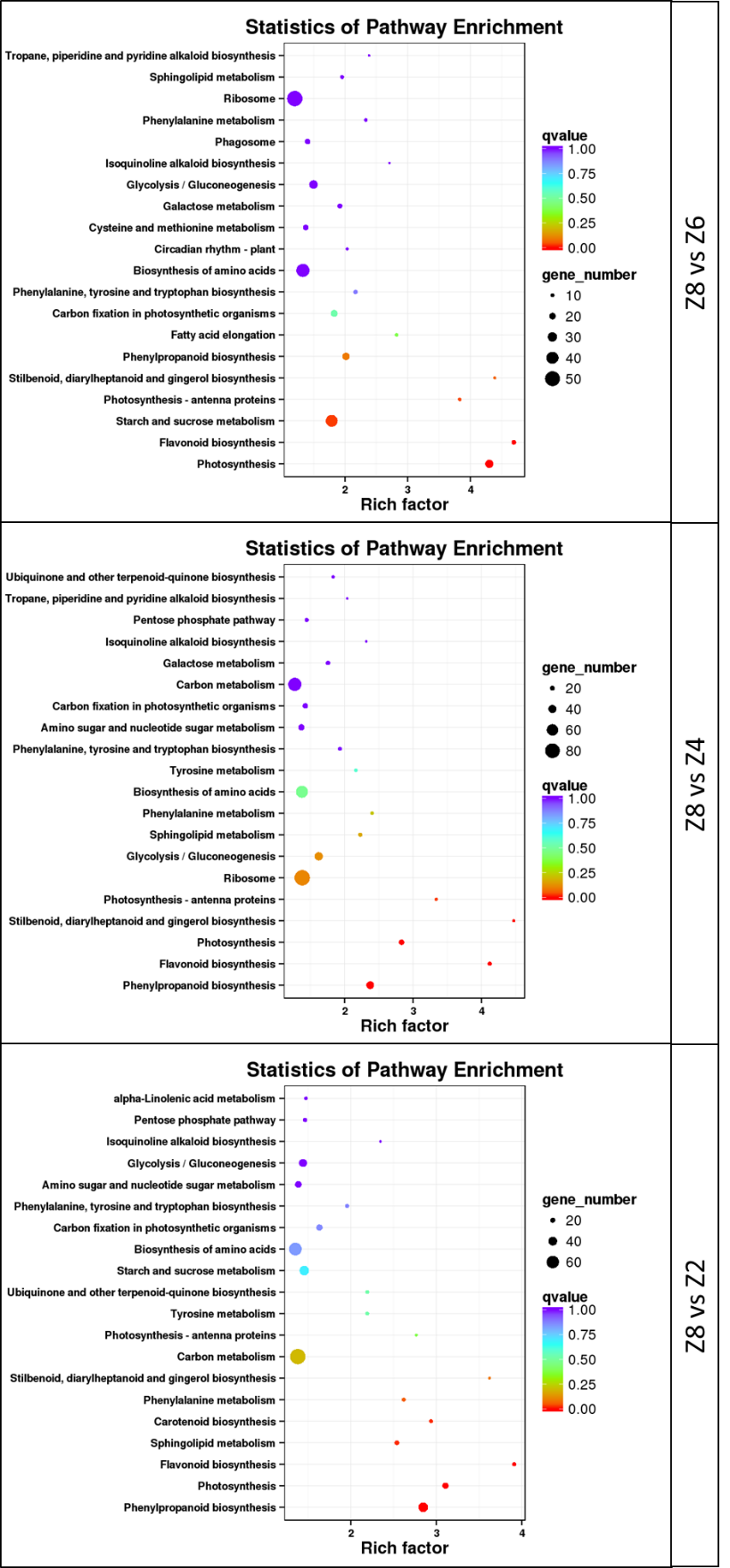


**Supplementary Figure 1.** KEGG pathway enrichment with differentially expressed genes between drought treated *P. kingianum* tubers. The soil water content was Z8 (80%), Z6 (60%), Z4 (40%), and Z2 (20%) of the maximum water holding capacity of the filed soil.
